# Preclinical research of dihydromyricetin for fibrotic diseases

**DOI:** 10.3389/fphar.2025.1691796

**Published:** 2025-12-03

**Authors:** Xiao-Chun Wang, Ping Mai, Zuo-Hui Yuan, Zhi-Sheng Qiu

**Affiliations:** 1 Department of Gastroenterology, Gansu Provincial Hospital, Lanzhou, China; 2 Department of Gastroenterology, The Eighth Affiliated Hospital, Southern Medical University (The First People’s Hospital of Shunde), Foshan, Guangdong, China; 3 Department of Surgical Oncology, Gansu Provincial Hospital, Lanzhou, China

**Keywords:** dihydromyricetin, fibrosis, anti-inflammatory, antioxidant, autophagy

## Abstract

Fibrosis is a pathological condition characterized by excessive deposition of extracellular matrix (ECM) components, leading to tissue scarring and progressive organ dysfunction. The effective treatment of fibrotic diseases remains a pressing challenge in medical research. Dihydromyricetin (DHM), a principal bioactive flavonoid derived from *Ampelopsis grossedentata*, exhibits diverse pharmacological activities, including anti-inflammatory, antioxidant, and autophagy-modulating effects. This comprehensive review systematically analyzes current research to elucidate the molecular mechanisms underlying DHM’s anti-fibrotic effects across various organ systems. Additionally, we assessed the compound’s chemical properties and toxicological profile. This review aims to advance the understanding of DHM’s therapeutic potential for fibrotic diseases, clarify associated molecular mechanisms, and highlight persistent challenges. We also propose new research directions to further decipher the mechanisms of action of this flavonoid, which may facilitate the development of novel therapeutic strategies for fibrotic diseases.

## 1 Introduction

Fibrosis is a pathological repair process that arises from physiological responses to chronic diseases and injuries, characterized by excessive accumulation of ECM components like collagen, fibronectin, and proteoglycans (Goetsch and Niesler, 2016). Normally, tissue repair involves coordination among inflammatory mediators, growth factors, and matrix remodeling enzymes to restore structural integrity (Winkler and Fowlkes, 2002; Jiang et al., 2007; Clarke et al., 2013). However, persistent harmful stimuli often lead to aberrant ECM deposition and fibrosis development. Fibrosis can affect multiple organs, including the heart, liver, kidneys, lungs, intestines, skin, and connective tissues, potentially compromising organ anatomy and function. According to the Global Burden of Disease Study 2019 (GDaI, 2020), fibrotic diseases accounted for 16.5% of global mortality in 1990, a figure that increased to 17.8% by 2019. When cancer was included within the category of fibrotic diseases, their overall contribution to global deaths rose from 28.7% in 1990 to 35.4% in 2019. These findings underscore the necessity of continued research into fibrotic diseases to develop effective anti-fibrotic therapies-a critical need that remains largely unmet.

**FIGURE 1:**
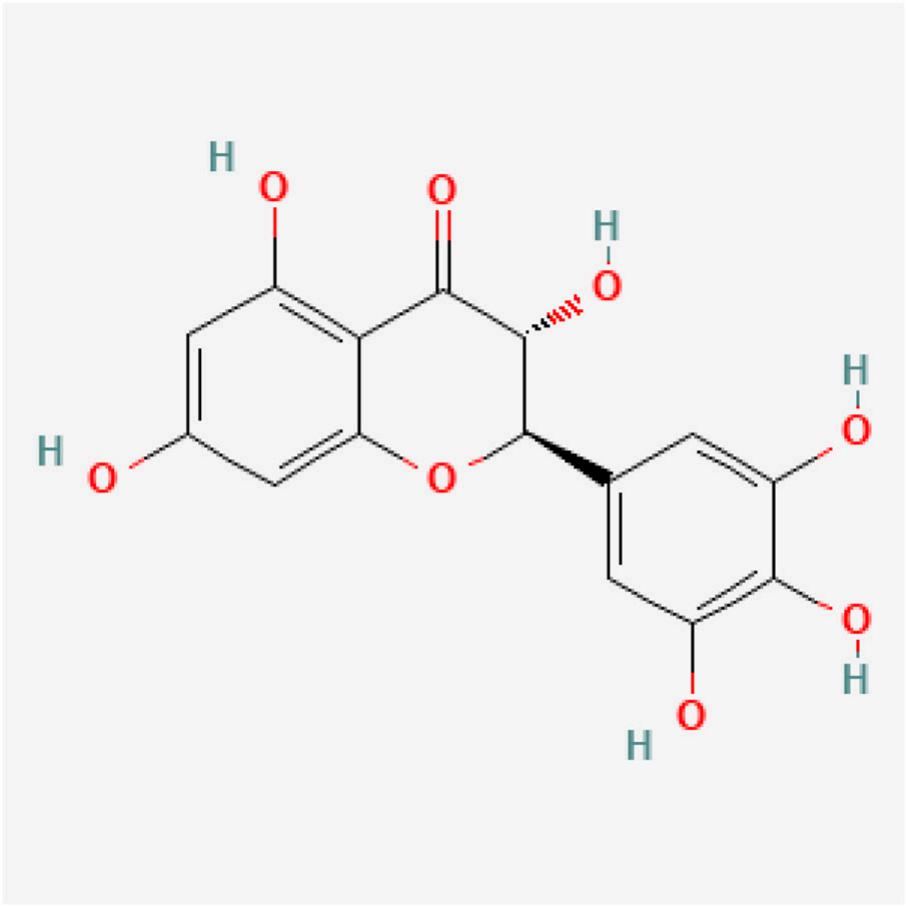
The chemical structure of DHM.

Dihydromyricetin (DHM; chemically defined as 3,5,7,3′,4′,5′-hexahydroxy-2,3-dihydroflavonol), also known as ampelopsin, is the primary active compound found in *Ampelopsis grossedentata* (Hand.-Mazz.) W.T.Wang (family Vitaceae). Its content in the dried leaves of *A. grossedentata* (commonly known as Tengcha) can account for up to 30% of the dry weight (Guo et al., 2019) (Figure 1). Recent studies have highlighted its diverse biological activities, such as anti-diabetes (Xiang et al., 2019), anti-hypertensive (Li Q. et al., 2017), anti-microbial (Shevelev et al., 2020), anti-atherosclerosis (Williams et al., 2015; Yang et al., 2020), anti-oxidative (Guo et al., 2019), anti-inflammatory (Zhou et al., 2017), and anti-carcinogenic (Tan et al., 2019) effects; Moreover, emerging evidence indicates that DHM exerts anti-fibrotic effects based on empirical evidence from preclinical studies, including *in vivo* animal models (e.g., rodent models of liver or lung fibrosis) and *in vitro* cell cultures (e.g., hepatic stellate cells or cardiac fibroblasts) (Table 1).

**TABLE 1:** The list of effects, mechanisms, concentrations, models of DHM in fibrotic disease.

Study/Years of publication	Effects	Mechanisms	Animal models/Subjects	Cell lines	Concentration/Time
Zhao et al. (2021)	Inhibiting the activities of ALT,AST,MDA and hepatocyte apoptosis; Increasing the levels of SOD and GSH	Inhibiting the expression of NF-κB signaling pathway; Decreasing hepatocyte apoptosis by regulating the PI3K/Akt signaling pathway	TAA-induced mice	NA	20 mg/kg and 40 mg/kg for 28 days
Zhou et al. (2021)	Decreasing ALT,AST, CoL-1α1, CoL-1α2,TIMP-1; Increasing the expression of LC3B-II; enhancing NK cell mediated killing of HSCs by increasing IFN-γ expression	Inducing autophagy and enhancing NK cell-mediated killing through the AhR-NF-κB/STAT3-IFN-γ signaling pathway	CCL_4_-induced mice	LX2 cells stimulated by TGF-β1	100 mg/kg once a daily for 6 weeks (for mice); 0,10, 30 and 50 μM for 2 h (for cells)
Li et al. (2022)	Regulating the differentiation of fibroblasts to myofibroblasts; Suppressing the abnormal migration, proliferation, and respiratory functions of myofibroblasts; Alleviating pulmonary fibrosis	Regulating the STAT3/p-STAT3/GLUT1 signaling pathway	BLM-induced mice	PMLFs stimulated by TGF-β1	300 mg/kg every day for 13days (for mice); 100,200,300 μM for 1h (for cells)
Xiao et al. (2021)	Inhibiting the infiltration of inflammation cells and the secretion of inflammation; Improving pulmonary function; Down-regulating the expression of α-SMA and fibronectin; Inhibiting the migration and activation of myofibroblasts and extracellular matrix production	Down-regulating the TGF-β1/Smad pathway	BLM-induced mice	Mlg cells stimulated by TGF-β1	50,100,200 mg/kg everyday for 7days (for mice); 10,20, 40 μM for 24h (for cells)
Song et al. (2017)	Decreasing cellular reactive oxygen species production and MDA level; Increasing the SOD activity and T-AOC; Suppressing p22phox, enhancing antioxidant SOD and Trx expression	NA	NA	Cardiac fibroblasts stimulated by Ang II	20, 40, 80 μM for 4 h, 12 h or 24 h
Wu et al. (2017)	Attenuating oxidative stress (MDA, SOD, and GSH-Px), reducing the levels of inflammation factors (IL-6, TNF-α), alleviating pathological changes, improving mitochondrial function inhibited cardiac apoptosis and restoring autophagy	Activating the AMPK/ULK1 and restoring autophagy	STZ-induced mice	NA	100 mg/kg/d for 14 weeks
Guo et al. (2020)	Attenuating the RIF and increasing autophagy in DN rat model; Inhibiting HG-induced fibrosis and promoting autophagy	Inhibiting the miR-155-5p/PTEN and PI3K/AKT/mTOR signaling pathway	STZ-induced rat	NRK-52E cells induced by HG	100 mg/kg/d for 10 weeks
Wang et al. (2024)	Alleviating intestinal inflammation, inhibiting the intestinal fibrosis and inducing the activation of autophagy	Downregulating the PI3K/AKT/mTOR signaling pathway	Mice fed with 2.5% DSS	CCD-18Co cells stimulated by TGF-β1	62.5,125,250 mg/kg everyday for 60 days (for mice); 12.5,25,50,100 μM for 24h (for cells)
Wu et al. (2019)	Reducing the erythema and swelling in the paws of CIA rats; Inhibiting the proliferation, migration and inflammation and increasing IL-1β-induced FLSs apoptosis	Suppressing the NF-κB signaling	Rats were immunized with bovine collage II	FLSs treated with interleukin IL-1β	5, 25 and 50 mg/kg by ip every other day for 5 weeks (for mice);

This review summarizes current knowledge on the pharmacology of DHM, with a focus on the pathological processes of fibrotic diseases and relevant experimental models. We suggest some research areas where this compound may exhibit beneficial effects on fibrotic diseases.

## 2 Pharmacokinetics of DHM *in vivo*

DHM contains multiple functional groups, including hydroxyl and carbonyl moieties, which contribute to its antioxidant and anti-inflammatory properties (Ondrejicková et al., 2006; Li et al., 2016; Liu et al., 2018). However, the phenol hydroxyl groups also confer chemical instability, leading to transformations including oxidation, hydrolysis, ring fission and reduction, all contributing to metabolite formation (Xiang et al., 2017). DHM demonstrates stability in weak acidic solutions (pH1.2-4.6) but undergoes degradation under basic conditions with partial degradation observed at pH 6.0 (Ruan et al., 2005). The poor solubility of DHM represents another critical factor limiting its bioavailability. Specifically, DHM exhibits solubilities of 0.2–0.32 mg/mL in cold water (25 °C), 20 mg/mL in hot water (80 °C), and 170 mg/mL in ethanol (25 °C) (Ruan et al., 2005). Following oral administration, DHM rapidly distributes to the gastrointestinal tract but demonstrates poor systemic absorption, with an oral bioavailability of approximately 4.02% (Liu and Wang, 2017). This indicates that only a small fraction of the administered dose reaches systemic circulation compared to intravenous delivery.

In mice orally administered DHM at 100 mg/kg, peak plasma concentrations (Cmax) reached 81.3 ng/mL and 107 ng/mL, with corresponding peak times of 36.7 min and 160 min, and elimination half-lives of approximately 288 min and 367 min, respectively (Tong et al., 2015). The elimination of DHM occurs relatively rapidly, with complete clearance within approximately 12 h, suggesting minimal potential for long-term accumulation (Fan et al., 2017). DHM uptake and transport are optimized under conditions of decreasing pH (from 8.0 to 6.0), exhibiting both time- and concentration-dependent characteristics. Metabolic profiling identified eight metabolites in urine and feces, but not in plasma, generated through various pathways including glucuronidation, reduction, dihydroxylation, sulfation, and methylation (Fan et al., 2017). Another study (Zhang et al., 2007) detected seven metabolites in urine and feces (four in rat urine and three in rat fecal specimens), representing five distinct metabolic pathways: glucuronidation, reduction, dihydroxylation, isomerization, and methylation. Gut microbiota significantly influences DHM pharmacokinetics. Fecal microflora metabolizes DHM into three primary metabolites via dihydroxylation and reduction pathways, with dihydroxylation representing the dominant metabolic route (Fan et al., 2018). Fan et al. (2018) demonstrated that pseudo-germ-free rats exhibited significantly higher Cmax values compared to control animals, indicating substantial involvement of intestinal flora in DHM metabolism.

Although comprehensive toxicity studies of DHM remain limited, existing evidence suggests low acute toxicity. Zhou et al. (2001) evaluated the safety profile of *A. grossedentata* extract (Teng tea), which contains high DHM levels, through acute toxicity, genetic toxicity tests and 90-day feeding tests. These investigations demonstrated toxicological safety and enhanced immunological function in mice. Zhang et al. (2014) reported no significant adverse effects in mice at doses ranging from 150 mg/kg to 1.5 g/kg, while Su et al. (2009) conducted acute toxicity tests showing minimal DHM toxicity, with a maximum tolerated oral dose of 5.0 g/kg body weight in rats. These pharmacokinetic characteristics highlight the necessity for developing advanced formulation strategies to improve DHM delivery efficiency. Furthermore, additional *in vivo* and *in vitro* studies are essential to establish comprehensive safety profiles and optimize therapeutic applications.

## 3 DHM's pan-assay interference substances (PAINS) properties

We explicitly acknowledge that DHM contains structural features (catechol group, polyphenolic structure) that classify it as a potential PAINS compound. These properties may contribute to false-positive results through various mechanisms including redox cycling, metal chelation, non-specific protein binding, and membrane disruption (Baell et al., 2010; Bolz et al., 2021). Therefore, we have implemented the following strategy in our review:

Evidence Hierarchy System: We categorized studies based on their experimental evidence level (Table 2):

Level 1: *In vitro* only studies (interpreted with caution)
Level 2: *In vitro* with comprehensive counter-screening assays
Level 3: *In vivo* validation with mechanistic studies
Level 4: Multiple animal models and clinical evidence

**TABLE 2:** Evidence level assessment of DHM’s anti-fibrotic effects.

Organ system	Evidence level	Key studies	PAINS mitigation strategies	Reliability score
Cardiac	Level 2	Clarke et al. (2013)	Comprehensive *in vitro* counter-screening	Moderate (Requires *in vivo* validation)
Hepatic	Level 3	GDaI, 2020; Guo et al. (2019)	Orthogonal *in vivo* and *in vitro* models	High
Pulmonary	Level 3	Xiang et al. (2019)	Orthogonal *in vivo* and *in vitro* models	High
Renal	Level 3	Li et al. (2017a), Shevelev et al. (2020)	Orthogonal *in vivo* and *in vitro* models	High
Intestinal	Level 3	Williams et al. (2015)	Orthogonal *in vivo* and *in vitro* models	High
Dermal/Connective Tissue	Level 3	Yang et al. (2020)	Orthogonal *in vivo* and *in vitro* models	High

Evidence Level criteria: Level 2 = *In vitro* studies with rigorous counter-screens; Level 3 = *In vivo* studies with supporting mechanistic insights.

## 4 Antioxidant effects of DHM

Free radicals, characterized as highly reactive molecules, initiate oxidative stress that subsequently induces cellular damage and contributes to the pathogenesis of various diseases, including cancer and neurodegenerative disorders (Sander et al., 2004; Khan et al., 2016; Houldsworth, 2024). Accumulating evidence indicates that elevated levels of reactive oxygen species (ROS) promote systemic oxidative stress, resulting in molecular and cellular alterations that drive the development of tissue fibrosis (Murrell, 1993). Experimental investigations have demonstrated that DHM effectively reduces intracellular ROS levels in cellular models, thereby conferring protection against oxidative damage. For instance, in HTR-8/SVneo trophoblast cells subjected to hydrogen peroxide-induced oxidative stress, DHM treatment significantly attenuated oxidative injury, as evidenced by reduced levels of lipid peroxidation and protein oxidation biomarkers (Bruić et al., 2024).

The antioxidant properties of DHM are mediated through dual mechanisms: direct scavenging of oxygen free radicals and activation of the Nrf2 signaling pathway, which enhances the expression of cytoprotective enzymes including glutathione peroxidase (GPx), catalase, and heme oxygenase-1 (HO-1). In a nutritional intervention study utilizing growing-finishing pigs, dietary supplementation with DHM substantially improved systemic antioxidant capacity, demonstrated by enhanced total antioxidant capacity (T-AOC), elevated catalase (CAT) and glutathione peroxidase (GSH-Px) activities, and reduced malondialdehyde (MDA) concentrations. Complementary molecular analyses revealed DHM-mediated upregulation of HO-1 and NQO1 protein expression, accompanied by promoted nuclear translocation of Nrf2 and phosphorylation of ERK (Wei et al., 2022). Beyond these fundamental antioxidant mechanisms, DHM modulates multiple signaling pathways implicated in oxidative stress responses, including the MAPK (Jiang et al., 2014) and PI3K/Akt pathway (Yang et al., 2024), which collectively participate in the transcriptional regulation of detoxifying and antioxidant enzymes. Substantial experimental evidence confirms that DHM activates the ERK/Nrf2/HO-1 signaling axis, thereby amplifying cellular antioxidant responses while concurrently suppressing inflammatory processes (Wei et al., 2022; Hu et al., 2018; Luo et al., 2017). Additionally, DHM influences the nuclear factor-kappa B (NF-κB) pathway, potentially reducing the production of pro-inflammatory cytokines and further reinforcing its cytoprotective function against oxidative damage (Sukhovskaya et al., 2023). Through coordinated regulation of these interconnected signaling networks, DHM not only safeguards cellular integrity against oxidative stress but also promotes the maintenance of cellular homeostasis. These comprehensive mechanisms underscore the significant potential of DHM in mitigating oxidative stress and its implications for the prevention and treatment of fibrotic diseases.

## 5 Anti-inflammatory effects of DHM

Research has demonstrated that DHM exerts anti-inflammatory effects through multiple molecular mechanisms. In lipopolysaccharide (LPS)-stimulated macrophages, DHM potently inhibits the activation of the NF-κB signaling pathway, thereby reducing the secretion of key pro-inflammatory cytokines, including tumor necrosis factor-α(TNF-α) and interleukin-6 (IL-6) (Sukhovskaya et al., 2023). Furthermore, DHM exhibits bidirectional regulatory capacity over macrophage polarization attenuating the pro-inflammatory M1 phenotype while concurrently promoting the polarization of anti-inflammatory M2 macrophages (Zhou et al., 2022). Furthermore, DHM demonstrates significant modulatory activity in mast cell, effectively suppressing degranulation and regulating the release of inflammatory mediator (Xiao et al., 2021). This multimodal immunomodulatory mechanism highlights DHM’s therapeutic potential in resolving inflammation and promoting tissue repair. Further investigations have revealed that DHM’s anti-inflammatory activities involve orchestrated regulation of complex signaling networks. In viral infection models, DHM interacts specifically with Toll-like receptor 9 (TLR9) to suppress activation of inflammatory transcription factors, thereby reducing cytokine release during herpes simplex virus infection (Takeda et al., 2011; Sauter et al., 2014; Zhou et al., 2020). Additionally, DHM attenuates influenza virus-induced immunopathology by inhibiting the TLR3 signaling pathway (Zhou et al., 2020). In metabolic contexts, DHM activates the Ca^2+^-CaMKK-AMPK pathway, improving insulin sensitivity and ameliorating inflammation-driven insulin resistance (Hou et al., 2021). The compound also manifests significant anti-inflammatory effects through modulation of the JAK/STAT signaling pathway in multiple disease models (Song et al., 2024). The significance of these broad anti-inflammatory mechanisms is particularly evident in the context of fibrosis pathogenesis, wherein chronic inflammation acts as a primary instigator and sustained driver. The persistent inflammatory milieu, characterized by the infiltration of innate and adaptive immune cells and their release of pro-fibrotic mediators, orchestrates the activation of resident fibroblasts and their differentiation into collagen-producing myofibroblasts, ultimately leading to excessive ECM deposition and organ dysfunction. Thus, the ability of DHM to target these key upstream inflammatory pathways presents a crucial therapeutic strategy for mitigating fibrosis progression. Collectively, these findings underscore the considerable therapeutic potential of DHM as a multi-targeted agent against various inflammatory diseases, highlighting its promise for the development of novel therapeutic strategies.

## 6 Anti-fibrosis effect of DHM

### 6.1 Effects of DHM in animal models of liver fibrosis

Liver fibrosis results from chronic injury caused by various stimuli including viral infections, alcohol consumption, cholestasis, autoimmune responses, and drug toxicity, leading to excessive deposition of fibrous connective tissue, architectural distortion, and ultimately functional impairment. The activation of hepatic stellate cells (HSCs) represents a central event in the pathogenesis of liver fibrosis (Zhang et al., 2016). Upon liver injury, quiescent HSCs transdifferentiate into proliferative, contractile, and fibrogenic myofibroblasts that secrete large amounts of ECM components and collagen, ultimately leading to fibrous scar formation and disruption of normal liver architecture (Tsuchida and Friedman, 2017). Alpha-smooth muscle actin (α-SMA) serves as a well-established marker of HSC activation, while transforming growth factor-β1 (TGF-β1) functions as the primary profibrogenic cytokine, with its signaling pathway being critically implicated in numerous liver diseases (Wynn and Ramalingam, 2012).

Oxidative stress not only causes persistent hepatic damage but also activates key signaling pathways such as TGF-β1/Smad and NF-κB, collectively promoting the development and progression of liver fibrosis (Gao et al., 2021). Zhao et al. (2021) demonstrated that in a thioacetamide (TAA)-induced liver fibrosis model in ICR mice, daily administration of DHM (20–40 mg/kg) via oral gavage reduced inflammatory cell infiltration in liver lobules, attenuated collagen deposition, and diminished the liver weight increase associated with fibrosis. Furthermore, DHM treatment enhanced the activities of superoxide dismutase (SOD) and glutathione peroxidase (GSH-Px), decreased serum levels of alanine aminotransferase (ALT) and aspartate aminotransferase (AST), and resulted in a dose-dependent reduction in hepatic malondialdehyde (MDA) content. These findings indicate that DHM alleviates TAA-induced oxidative stress and liver injury, exhibiting hepatoprotective effects. Further investigations revealed that DHM effectively suppresses TGF-β1 secretion and α-SMA expression, inhibiting HSC activation and thereby attenuating liver fibrosis progression. Additionally, DHM ameliorates liver fibrosis by inhibiting NF-κB signaling activation, reducing the release of inflammatory cytokines including TNF-α and interleukin-1β (IL-1β), and increasing the expression of anti-apoptotic proteins Bcl-2 and Bcl-XL.

Natural killer (NK) cells play a crucial role in liver fibrosis regression by producing interferon-γ (IFN-γ), which acts through the JAK/STAT signaling pathway to block TGF-β signaling and exert antifibrotic effects (Jeong et al., 2006). NK cells can also directly kill activated HSCs and promote their apoptosis (Fasbender et al., 2016). Studies have shown that DHM (100 mg/kg/d) reduces serum ALT and AST levels in mice, ameliorating liver inflammation and fibrosis. *In vitro* experiments using the human hepatic stellate cell line LX-2 demonstrated that pre-incubation with DHM (at concentrations >30 μmol/L) significantly suppressed collagen type I (Col-I) and α-SMA protein expression. Mechanistic studies confirmed that DHM activates both NF-κB and JAK/STAT3 signaling pathways to stimulate IFN-γ production, induce autophagy, and enhance NK cell-mediated cytotoxicity, thereby inhibiting HSC activation and attenuating liver fibrosis progression (Zhou et al., 2021). Ma’s team (Ma et al., 2019)further demonstrated that DHM inhibits HSC activation by modulating the SIRT1/TGF-β1/Smad3 signaling and autophagy pathways, effectively attenuating CCL_4_-induced hepatic fibrosis.

### 6.2 Effects of DHM in animal models of pulmonary fibrosis

Multiple etiological factors contribute to pulmonary fibrosis pathogenesis, including pharmaceutical agents, particulate matter exposure, viral infections, and chronic inflammatory stimuli. Pulmonary fibrosis is characterized by irreversible alveolar epithelial cell injury, aberrant fibroblast proliferation, and excessive ECM deposition, leading to compromised pulmonary function, elevated airway resistance, and respiratory insufficiency. These pathological changes severely compromise quality of life and may ultimately prove fatal (Thannickal et al., 2004).

Persistent inflammatory stimuli promote excessive division and proliferation of pulmonary fibroblasts, accelerating their differentiation into myofibroblasts and resulting in pathological ECM accumulation, thereby driving pulmonary fibrosis progression (Tao et al., 2021). Experimental studies have demonstrated the therapeutic potential of DHM in pulmonary fibrosis management. In a bleomycin (BLM)-induced lung injury murine model, DHM administration significantly attenuated pulmonary inflammation and fibrosis. Specifically, DHM effectively inhibited fibroblast-to-myofibroblast transition, consequently reducing pulmonary collagen deposition and fibrotic lesion formation. Furthermore, studies indicate that DHM exerts its anti-fibrotic effects through modulation of the STAT3 signaling pathway, which plays an indispensable role in fibroblast activation and ECM production (Li et al., 2022). Notably, studies revealed that DHM significantly suppresses inflammatory cell infiltration in pulmonary tissues and improves respiratory function, representing a crucial mechanism for alleviating pulmonary fibrosis (Xiao et al., 2021). Mechanistic investigations established that DHM mediates its protective effects primarily through reduction of pro-fibrotic markers and inflammatory cytokines, which effectively suppresses fibrotic responses and creates a favorable microenvironment for lung tissue repair (Matouk et al., 2023).

TGF-β1 induces pulmonary fibroblast activation and regulates the TGF-β1/Smad signaling axis, thereby promoting pulmonary fibrosis development (Chaikuad and Bullock, 2016). Pharmacological modulation of the TGF-β1/Smad pathway represents a promising therapeutic target for pulmonary fibrosis intervention. Xiao et al. (2021) established a BLM-induced pulmonary inflammation and fibrosis model in C57/BL6 mice, with experimental groups receiving DHM at doses of 50–200 mg/kg/day. DHM treatment significantly ameliorated pulmonary inflammation and fibrosis compared to control animals. Additional investigations demonstrated that DHM reduces early inflammatory factor secretion and inhibits inflammatory cell infiltration, thereby substantially alleviating pulmonary inflammation and improving lung function, demonstrating its potential as a therapeutic agent. *In vitro* studies further confirmed that DHM significantly inhibits fibroblast migration and ECM production through regulation of the TGF-β1/Smad signaling pathway, suggesting that DHM may effectively ameliorate pulmonary inflammatory and fibrotic pathologies.

### 6.3 Effects of DHM in animal and cell models of cardiac fibrosis

Multiple cardiovascular diseases, including hypertension, cardiomyopathy, and myocardial infarction, contribute to myocardial fibrosis pathogenesis. This condition is characterized by aberrant cardiac fibroblast proliferation and excessive ECM deposition, ultimately compromising normal cardiac architecture and precipitating functional impairments, including heart failure, arrhythmias, and even sudden cardiac death (González et al., 2018).

Research has confirmed that oxidative stress plays a pivotal role in the initiation and progression of myocardial fibrosis (Guillory et al., 2018). NADPH oxidase (NOX),a key enzymatic source of reactive oxygen species in the cardiovascular system (Holterman et al., 2015), exhibits upregulated expression and dysregulated activity under pathological conditions. Such alterations exacerbate oxidative damage to cardiac fibroblasts and promote ECM accumulation, thereby driving myocardial fibrosis progression (Wang et al., 2021). Therefore, targeting oxidative stress and its associated molecular components-including proteins, cytokines, and signaling pathways-represents a crucial therapeutic strategy for myocardial fibrosis intervention. Song et al. (2017) established a cardiac fibroblast proliferation model in Sprague-Dawley (SD) rats using angiotensin II (Ang II), with subsequent DHM intervention. The results demonstrated that DHM inhibited cardiac fibroblast proliferation in both concentration-dependent (20, 40, 80 μmol/L) and time-dependent (4, 12, 24 h) manners. Specifically, DHM suppressed the expression of Col-I, Col-III, and α-SMA.

Furthermore, administration of a high concentration (320 μmol/L) of DHM demonstrated no significant cytotoxicity, thereby eliminating potential confounding effects on fibroblast proliferation. Additionally, DHM significantly reduced myocardial MDA levels and nitric oxide (NO) concentrations, while enhancing superoxide dismutase (SOD) activity and T-AOC. These findings also indicated that DHM not only suppressed p22phox expression but also increased the levels of antioxidant proteins, including SOD and thioredoxin (Trx) in Ang II-stimulated myocardial fibroblasts (Suresh et al., 2015).

AMPK regulates SIRT3 expression, thereby attenuating myocardial fibrosis progression by reducing oxidative stress levels (Chen et al., 2017). Wu et al. (2017) established a streptozotocin (STZ)-induced diabetic cardiomyopathy (DCM) model in C57BL/6 mice and administered DHM (100 mg/kg/day) via oral gavage. The results demonstrated that myocardial fibrosis was significantly attenuated and cardiac function was improved in DHM-treated mice compared to controls, concomitant with reduced myocardial MDA content and enhanced T-AOC. Further mechanistic investigations revealed that DHM upregulated SIRT3 expression while downregulating receptor-interacting protein kinase 3 (RIPK3) in myocardial tissues. Additionally, DHM enhanced the expression of antioxidant proteins and regulated the AMPK/SIRT3 signaling pathway, thereby mitigating oxidative stress-mediated myocardial damage and alleviating fibrotic remodeling through coordinated modulation of the AMPK/SIRT3 signaling axis.

### 6.4 Effects of DHM in animal models of renal fibrosis

Renal fibrosis represents a common pathological endpoint in numerous chronic kidney diseases, including primary glomerulonephritis, diabetic nephropathy, and obstructive nephropathy, ultimately progressing to end-stage renal disease (ESRD). The primary pathological features encompass epithelial-mesenchymal transition (EMT) of renal tubular epithelial cells, excessive deposition of ECM in glomerular and interstitial compartments, and persistent fibroblast activation, culminating in tubular injury, reduced glomerular filtration rate, and architectural and functional impairment of renal tissue (Nogueira et al., 2017).

Accumulating evidence has established TGF-β1 as a pivotal cytokine driving renal fibrogenesis. TGF-β1 activates the TGF-β1/Smads signaling pathway, promoting EMT-mediated transformation of renal tubular cells into myofibroblasts, thereby exacerbating fibrotic processes (Ma and Meng, 2019). Diabetic kidney disease (DKD) represents a major etiology of renal fibrosis, wherein hyperglycemic conditions enhance TGF-β1 activity and upregulate TGF-β1 mRNA expression, stimulating fibroblast proliferation and ECM deposition that accelerate renal interstitial fibrosis (RIF) progression (Zhao et al., 2020). Liu ZL. et al. (2019) established a DKD model in SD rats using STZ and administered DHM at doses of 125, 250, and 500 mg/kg/day via oral gavage for 12 weeks. Post-treatment assessment revealed significant reductions in 24-h urinary protein excretion, blood urea nitrogen (BUN), and serum creatinine (Scr) levels compared to control animals. Histopathological analysis revealed that DHM treatment ameliorated glomerular basement membrane thickening and collagen fiber proliferation. These findings indicate DHM’s nephroprotective effects against DKD-induced renal damage and fibrosis. Further mechanistic studies demonstrated that DHM downregulated TGF-β1 and Smad2 protein expression while upregulating Smad7, suggesting inhibition of TGF-β1/Smads pathway activation. Li JL. et al. (2017) investigated DHM in high glucose-stimulated mesangial cells (MCs), revealing that DHM binds directly to Smad2, inhibits its phosphorylation, and suppresses TGF-β/Smads signaling activation, thereby improving DKD-associated glomerulosclerosis.

Peroxisome proliferator-activated receptor gamma (PPARγ) regulates phosphatase and tensin homolog (PTEN), consequently negatively modulating the PI3K/AKT pathway and attenuating renal fibrosis (Cong et al., 2020). The PI3K/AKT/mTOR pathway represents a key signaling cascade mediating fibrotic processes in various renal pathologies, including diabetic nephropathy and acute kidney injury. This pathway critically controls renal fibrogenesis through PI3K/AKT/mTOR signaling regulation (Dou et al., 2019).

MiR-155-5p is predominantly expressed in mesangial cells, glomerular endothelial cells and renal tubules, highlighting its pathological relevance in diabetic nephropathy (DN). Elevated miR-155-5p levels in renal tissues of DN patients promote RIF progression and inhibit autophagy in renal interstitial fibroblasts, with expression levels correlating with DKD severity (Klimczak et al., 2017). Guo et al. (2020) established a DN model in 6-week-old SD rats, treating them with 100 mg/kg/day DHM for 10 weeks. Immunohistochemical analysis demonstrated elevated expression of Col-IV and α-SMA in the DN group compared to normal controls, while DHM treatment attenuated these increases. Notably, DHM not only reduced miR-155-5p expression but also enhanced PTEN expression.

Mechanistic investigations revealed that DHM primarily inhibits RIF progression and promotes renal interstitial cell autophagy by suppressing miR-155-5p expression and modulating both PI3K/AKT/mTOR and miR-155-5p/PTEN signaling pathways. Autophagy induction constitutes a crucial protective mechanism against cellular damage, potentially ameliorating renal impairment in human kidney diseases (Hartleben et al., 2010). The PI3K/AKT/mTOR signaling pathway represents a classical regulatory mechanism associated with autophagy (Sun et al., 2013). In this study, DHM promoted high glucose (HG)-induced autophagy in NRK-52E cells and reduced renal fibrosis (Guo et al., 2020). Additionally, studies indicate that DHM alleviates oxidative stress by enhancing expression of antioxidant proteins, particularly nuclear factor erythroid 2-related factor 2 (Nrf2), which is crucial for cellular protection (Wang et al., 2020). DHM inhibits Smad3 activation, an essential component of TGF-β signaling, thereby preventing fibroblast activation and collagen production (Wang et al., 2020).

MiR-34a serves as a transcriptional target of p53, regulating cell cycle progression and apoptosis (Slabáková et al., 2017). Klotho, primarily expressed in renal tubular epithelial cells, functions as an important endogenous inhibitor of renal tubulointerstitial fibrosis. Initially identified as an anti-aging gene, Klotho plays significant roles in protecting against acute and chronic kidney injury (Satoh et al., 2012). Liu Y. et al. (2019) demonstrated increased miR-34a expression in renal tubular epithelial cells of patients with renal fibrosis and unilateral ureteral obstruction (UUO) mice (Liu et al., 2018). Using miR-34a knockout mice, they confirmed that miR-34a promotes renal fibrosis by downregulating Klotho. *In vitro* experiments showed that TGF-β1-stimulated human renal tubular epithelial cells (HK-2) exhibited increased miR-34a and α-SMA expression along with enhanced collagen deposition. DHM treatment inhibited both p53/miR-34a/sirtuin 1 (SIRT1) and TGF-β1/Smads signaling pathways, upregulating Klotho expression and consequently attenuating renal fibrosis (Wang et al., 2020). Collectively, these findings suggest that DHM represents a promising therapeutic candidate for patients with chronic kidney disease and renal fibrosis.

### 6.5 Effects of DHM in animal models of intestinal fibrosis

Intestinal fibrosis represents a serious complication associated with Crohn’s disease (CD) and ulcerative colitis (UC). In this pathology, excessive accumulation of ECM leads to a progressive loss of intestinal tissue elasticity, resulting in luminal narrowing and a substantial decline in the patient’s quality of life. The pathophysiology of intestinal fibrosis is complex, involving EMT, activation of intestinal fibroblasts, and chronic inflammation. Inflammation is often seen as a precursor to fibrosis; however, evidence indicates that fibrotic progression can persist independently of active inflammation. TGF-β is a key driver of fibroblast activation and ECM production in this process. Additionally, the intestinal microbiota is implicated in the pathogenesis of intestinal fibrosis, whereby microbial dysbiosis can promote fibroblast activation and fibrotic reactions (Chen et al., 2021; Watanabe and Kamada, 2022).

Emerging research suggests that DHM alleviates intestinal fibrosis by inducing autophagy in colonic fibroblasts, thereby inhibiting their activation and proliferation (Wang et al., 2024). This effect is potentially mediated through the regulation of the PI3K/AKT/mTOR pathway, a pivotal pathway regulating autophagy and cell survival. By promoting autophagic flux, DHM inhibits fibroblast activation, thereby attenuating the progression of intestinal fibrosis (Wang et al., 2024). This protective property positions DHM as a potential therapeutic agent for conditions characterized by intestinal fibrosis.

### 6.6 Effects of DHM in animal models of other fibrotic diseases

Rheumatoid arthritis (RA) is a chronic autoimmune disorder that primarily affects multiple joints. Joint impairment facilitates the infiltration of numerous immune cells into the synovial tissue, leading to synovial swelling, joint deformity, and progressive functional impairment (McInnes and Schett, 2011). Under physiological conditions, synovial fibroblasts maintain joint homeostasis by providing nutritional support and lubrication. However, upon immune cell infiltration, synovial fibroblasts undergo pathological proliferation and migration, contributing to progressive destruction of bone and articular cartilage. Consequently, targeting synovial fibroblasts and modulating joint inflammation represent promising strategies for RA therapy. Wu et al. (2019) established a collagen-induced arthritis (CIA) rat model to investigate RA pathogenesis. DHM was administered intraperitoneally at doses of 5, 25, and 50 mg/kg every other day for 5 weeks. DHM treatment significantly ameliorated clinical signs, including paw erythema and edema, in RA-induced rats. Histopathological examination of knee joints and analysis of peripheral blood cytokine levels further confirmed the potent anti-arthritic effects of DHM, demonstrating its ability to mitigate synovitis and systemic inflammatory responses. Mechanistically, DHM suppressed the phosphorylation of IκB kinase (IKK) and IκBα, significantly reducing IL-1β-induced nuclear translocation of NF-κB in fibroblast-like synoviocytes (FLSs).

Hypertrophic scarring (HPS) is characterized by dysregulated wound healing, featuring excessive ECM deposition and aberrant fibroblast activity. HPS tissue is predominantly composed of fibroblasts and collagen types I and III (Col-I, Col-III), commonly resulting from burns, trauma, or surgical interventions. Fibroblasts in HPS exhibit elevated levels of activin receptor-like kinase 5 (ALK5) compared to normal skin tissues (Tsujita-Kyutoku et al., 2005). The TGF-β/Smad signaling pathway is critically involved in the pathogenesis of proliferative scars, wherein Smad2 and Smad3 proteins bind to the TGF-β type I receptor (ALK5) and undergo phosphorylation, thereby activating downstream fibrotic responses. Ye et al. (2019) established a mechanical traction-induced HPS model in mice. Subsequent daily subcutaneous injections of approximately 50 μmol/L DHM for 14 days significantly reduced scar area, cross-sectional dimensions, cellular proliferation index, and collagen density. Further investigations revealed that DHM selectively inhibits ALK5 within the TGF-β/Smad pathway, leading to downregulation of Smad2 and suppression of Smad3 phosphorylation. These findings highlight the therapeutic potential of DHM for HPS management.

## 7 Conclusions and future prospects

**FIGURE 2:**
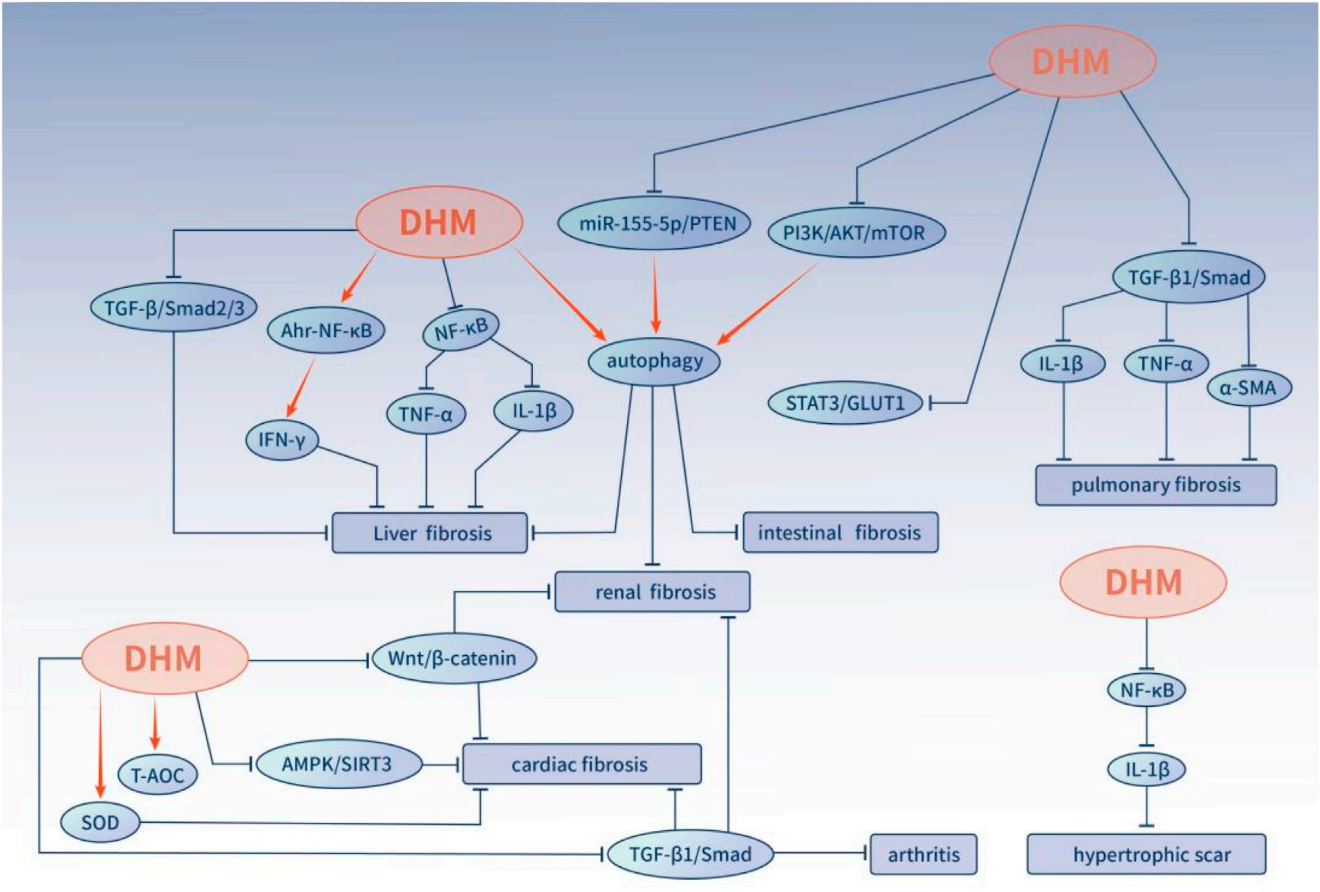
The mechanism and signaling pathway of DHM in various fibrotic diseases. Abbreviations: DHM,Dihydromyricetin; IL-1β,Interleukin-1β; AKT, protein kinase B; PI3K,Phosphatidylinositol 3-kinase; TNF-α, tumor necrosis factor-α; AMPK, Adenosine 5′-monophosphate activated protein; NF-κB, Nuclear factor-κB; STAT, signal transducer and activator of transcription; T-AOC, total antioxidant, capacity; SOD, superoxide dismutase; IFN-γ,interferon-γ.

This review systematically summarizes the mechanisms of DHM against fibrotic diseases, with particular emphasis on its anti-inflammatory, antioxidant, and autophagy-modulating properties (Figure 2). Although accumulating evidence suggests the anti-fibrotic potential of DHM, its pan-assay interference (PAINS) behavior urge cautious interpretation of existing data, particularly from studies relying solely on *in vitro* models, which may overestimate its target-specific efficacy.

To better establish the therapeutic relevance of DHM, future studies should focus on the following key directions:

- Employing advanced target engagement assays to confirm mechanistic specificity;
- Incorporating counter-screening strategies to exclude PAINS-related artifacts;
- Prioritizing *in vivo* validation using dosing regimens guided by pharmacokinetic studies;
- Investigating active metabolites of DHM that retain efficacy without PAINS-associated liabilities.

Moreover, although current evidence is largely derived from animal and cellular studies, clinical evidence remains limited. To advance the translational progress of DHM, the following approaches warrant priority:

- Conducting rigorously designed clinical trials to validate preclinical observations;
- Overcoming critical pharmacokinetic challenges, including low oral bioavailability and suboptimal intestinal absorption.

In conclusion, DHM demonstrates promising anti-fibrotic properties supported by consistent *in vivo* outcomes across multiple organ systems. However, future efforts must rigorously address its PAINS characteristics and accelerate clinical translation to fully realize its therapeutic potential.

## Abbreviations

DHM, Dihydromyricetin; ECM, extracellular matrix; ROS, reactive oxygen species; GPx, glutathione peroxidase; HO-1, heme oxygenase-1; T- AOC, total antioxidant capacity; CAT, catalase; GSHPx, glutathione peroxidase; MDA, malondialdehyde; LPS, lipopolysaccharide; HSCs, hepatic stellate cells; TGF-β, transforming growth factor-β; MMP1, matrix metalloproteinase 1; α-SMA, alpha-smooth muscle actin; SOD, superoxide dismutase; UUO, ureteral obstruction; CD, Crohn’s disease; UC, ulcerative colitis; EMT, epithelial mesenchymal transition; RA, Rheumatoid arthritis; FLSs, fibroblast-like synoviocytes; HPS, Hypertrophic scarring.
